# Supplementary material for: Neural and cardiorespiratory responses in vocalization and slow breathing: Contrasting brain and autonomic responses
Source: PLoS One. 2026 Apr 29;21(4):e0345719. doi: 10.1371/journal.pone.0345719 (PMC13128120; doi:10.1371/journal.pone.0345719)
Supplement: Attachment — Submitted filename: Response to Reviewers.pdf [file pone.0345719.s002.pdf]

Response to Reviewers

Article: Neural and cardiorespiratory responses in vocalization and slow breathing: contrasting brain and autonomic responses

Dear editor and reviewers,

We hope this email finds you well. We appreciate the time and effort you have put into offering your honest feedback on the current research. You provided valuable insights and comments that significantly improved the quality of the article. We have addressed every point that was raised and provided a detailed response below.

Best regards,
The authors

Reviewer 1:

1. Please provide the rationale for the chosen sample size. Was a power analysis conducted to determine adequacy?

It is important to note that this report, as part of a larger study (including a report on the cardio-respiratory effects and a report on the phenomenological aspects), had exploratory objectives. The adequacy of the sample size was based on prior studies tackling exploratory topics in the realm of cardiorespiratory interactions, including previous papers from our team. Some examples of studies having around 20 participants are Spicuzza et al (2000) in Lancet, Bernardi et al (2001) in BMJ, Bernardi et al (2006) in Heart, Bernardi et al (2009) in Circulation.

The sample size aligns with the recommendation for exploratory research, particularly for within-subject designs, suggesting that "increasing the sample size to 12 participants made a profound difference in the width of confidence intervals for mean response, whereas increasing the sample size beyond 12 participants did not. They referred to this result as a “rule of 12” for continuous variables. We recommend at least 12 participants for pilot studies with primary focus of estimating average values and variability for planning larger subsequent studies. This size is quite practical for most early‐stage investigators to conduct within single centers while still providing valuable preliminary information" (Moore et al., 2011).

For clarity, the following was added to the Participants' section:
"Having exploratory purposes and a within-subjects design, this study aimed to have more than twelve participants, as previously recommended (Moore et al., 2011)."

2. Given the repeated nature of the measurements, a repeated measures ANOVA would be more appropriate than a two-way ANOVA

Similar to the previous paper focusing on cardio-respiratory effects (Bernardi et al., 2017), we conducted a 2x2 repeated measures ANOVA. This was implemented using Matlab's function **ranova** with the parameter 'WithinModel'. For more clarity, we updated the manuscript to reflect "2x2 repeated measures ANOVA".

3. Statistical analyses should be included to identify differentially activated brain clusters between the vocalization and breathing conditions. A cluster-based permutation test might provide a clearer picture of localized neural effects.

Like previous research (Luft & Bhattacharya, 2015), we used cluster permutation analysis to statistically assess the brain areas that differed between the baselines before the intervention and the baselines following each intervention. This is described in the "Statistical analyses" subsection of "Methods" (l.179-189). A direct comparison between vocalization and breathing conditions was not made. One reason was that, due to the experimental procedure, the vocalized conditions always occurred before their corresponding matched breathing (see Fig. 1), and thus the direct comparison between vocal and breathing could have introduced confounds. It was deemed more valuable to compare the effects of the interventions with the control, which was the 1-minute resting state before the intervention. This cluster permutation approach allowed us to characterize the brain areas activating following each intervention (see figures 3-5), and we were able to find contrasting effects between vocalization and breathing conditions.

4. Were the p-values reported (e.g., lines 268–270) Bonferroni corrected? If so, please specify the correction method used. If not, multiple comparisons should be properly controlled.

To correct for multiple comparisons, we used the less conservation Holm-Bonferroni method (see line 222). Instead of dividing all p-values by a constant factor as in Bonferroni, this method sorts the p-values from the smallest to the largest and progressively adjusts the significance levels, in our case. Because the significance levels are not constant as in Bonferroni, it is not practical to present corrected values. What we present in Table 2 are the statistical significance levels of each p-value, following correction. For instance, when comparing singing with the

baseline for HR, the p-value is 0.018, which is not statistically significant after Holm-Bonferroni correction.

5. Several physiological measures are listed in Table 1, but not all are discussed in the main text. Please ensure consistency by either elaborating on all measures or justifying their omission.

We appreciate the observation. To make it clearer for the reader, we have included a sentence in the results section, mentioning which measures are presented in Table 1 and which ones were omitted, for reference. LF and SDNN measures were included only as a reference, to be able to validate the current sample of 16 participants, compared to the previous publication (Bernardi et al., 2017) that used 20 participants (4 datasets were excluded due to issues with the EEG). Compared to the previous publication, HRV-RMSSD was a new measure and thus it was discussed more thoroughly.

6. Given the diverse physiological measures applied, some of which are interrelated, a principal component analysis (PCA) or clustering analysis could provide a more holistic view of how these variables interact.

We appreciate the suggestion and understand why PCA can be useful to capture the interactions between variables. However, since we do use standard measures (e.g., Task Force, 1996, and Laborde et al., 2017), we believe PCA would be outside the purpose of this study, especially given the small sample we had. Future meta-analyses may indeed consider PCA as a way of better characterizing HRV components.

7. The rationale behind Figures 8 and 9 is unclear. What specific hypotheses or findings do these figures aim to illustrate?

The rationale behind Fig. 8 is exploratory and examines the potential relationship between beta power and HRV-RMSSD across all conditions. As such, there were no specific hypotheses. Increases in beta power following the interventions are linked to decreases in RMSSD. This negative correlation is only indicative of possible future studies that may more closely target underlying mechanisms. As discussed (l.392-404), the beta power increases observed for all conditions align with the literature, as we would expect all experimental conditions to require attention and cognitive control, and beta power being related to attention and cognitive control (Lee et al., 2018 and Güntekin et al., 2013).
Regarding the relationship of beta power and HRV-RMSSD, we speculated that higher HRV-RMSSD may reflect a regulatory state bringing down the attentional resources needed for the task. This is akin to the reduced use of attentional resources for experts vs. non-experts. However, we are aware this is speculative and of the potential limitations of this study to provide

any conclusion in this regard, and thus we believe this is an interesting relationship that future research may address.

Fig. 9 simply aimed to illustrate a well-known negative correlation between HR and HRV-RMSSD (see Kazmi et al., 2016, as presented in the discussion). Fig. 9 shows that the relationship holds across all conditions.

8. The discussion on vocalization-induced relaxation requires further clarification. Notably, singing and toning appear to increase HR and lower HRV, both indicative of higher arousal (stress). Attention is linked to increased alpha waves—how do these changes in brain oscillations relate to the observed cardiac responses in different arousal/attentional states?

The present research finds that toning and singing both increase HR while not associated with significant increases or decreases in HRV-RMSSD (see discussion, l. 330-348). We had hypothesized that toning to increase HRV-RMSSD because it is associated with lower respiration frequencies. We explained the results by the presence of two opposing forces at play. First, a lower respiration frequency for toning (with a respiration frequency around 0.1Hz) would lead to HRV-RMSSD increases, consistent with prior research and the previous publication showing increases in HRV-SDNN and HRV-LF. We only saw reliable RMSSD increases when participants were breathing at the same frequency as toning. As expected, the condition in which participants were breathing at the same frequency as singing (about 0.2Hz) did not show RMSSD increases.
Second, vocalized conditions (toning and singing) implied physical activity, in contrast to breathing alone, which is known to increase arousal and HR. We believe these two opposing forces present a parsimonious explanation of the results. The first force (lower respiration frequency) would predict increases in HRV-RMSSD for toning and corresponding breathing. The second force (physical activity) would predict increases in HR and, because HR and HRV-RMSSD are inversely correlated, a decrease in HRV-RMSSD. This explanation is consistent with all the observed effects (see Fig. 7a and 7b).

We discuss the possible relationship between alpha waves and the HRV-RMSSD (l.346-360). Vocal conditions (toning and singing) show alpha power increases and no HRV-RMSSD changes, while slow breathing (breathing at 0.1Hz) shows increases in HRV-RMSSD but no changes in alpha power. We first describe how our results point against a relationship between alpha power and HRV-RMSSD, which is typically used as an index of vagal afferent tone (Laborde et al., 2017). This is remarkable because in slow breathing intervention studies, the observed profile involves increases in alpha power and increases in HRV-RMSSD. Slow breathing may thus increase vagal afferent tone, which is an important pathway in the parasympathetic nervous system, but this may not reliably lead to alpha power increases.

We then offer alternative explanations explaining the observed increases in alpha power (l. 361-370 and l. 371-391), the first based on physical exertion as a possible mechanism, and the second involving a sensory-motor pathway, including the making of vocal sounds and the sonic feedback, which, as discussed, may induce entrainment. Because of the nature of the study and for practical reasons, it did not allow further distinction of the relative contribution of motor and sensory pathways. Future work could, for instance, contrast vocalization and only listening to vocalization.

9. How does motor control differ between matched breathing and vocalization (toning/singing)? A discussion on the biomechanical and neural bases of phonation would help clarify this aspect.

We appreciate the input and believe that a more in-depth revision of the motor and auditory mechanisms in place would constitute great follow-up research to be made. Due to the exploratory nature of the current study (which, to our knowledge, is the first EEG study of toning), we deem it outside its scope.

1. Abbreviations should be defined upon first use.

We have updated the text to define abbreviations upon first use (HRV-RMSSD, HRV). Well-known abbreviations across science are omitted (ECG, EEG).

2. HRV Metrics Reporting: SDNN, LF, HF, and RMSSD were calculated, but only RMSSD results were presented. Please clarify why the other measures were omitted.

As explained in the Methods (l.223-227), we re-analyzed HRV-SDNN and HRV-LF to test for statistical differences in the datasets used in this analysis compared to previously published results (Bernardi et al., 2017). The current analysis had full datasets for 16 participants, while the previous results were based on 20 participants, with the difference due to issues in the EEG data. As reported, the differences were minimal, which helps position this analysis in the context of the broader study. However, the main goal of this re-analysis was to introduce HRV-RMSSD as an index of vagal afferent tone to the picture.

Reviewer 2

Observations
1. The study reports post-intervention increases in alpha and theta power, yet task-state EEG is excluded due to vocalization artifacts. Without neural data during the intervention, conclusions about mechanisms or entrainment effects remain speculative.

We thank the reviewer for the comment and agree that there are limitations to studying only the differences in the baselines. Focusing only on the baseline changes was a methodological choice, common in the literature. Although it is possible to directly study the intervention effects on the brain, here we discarded the analyses to avoid experimental noise. There are some reasons for this methodological choice. Firstly, the noise profile of the experimental conditions is likely to differ because of the clear differences in motion between vocal and breathing-only conditions. Secondly, signal processing methods to remove noise may also have intrinsic limitations, e.g., because there is a lack of an objective reference measure (Dasa Gorjan et al. 2022, J. Neural Eng. 19 011004). Future work should address the limitations of this study.

2. Vocalization inherently involves sensorimotor feedback loops (auditory-motor coupling, efference copy, etc.), which are not considered. Without controlling for auditory feedback, it's impossible to separate proprioceptive vocal-motor activity from auditory-induced entrainment effects. No analysis of vocal acoustic features (pitch, loudness, variability) was conducted, yet these could modulate arousal and autonomic effects.

We appreciate the feedback and agree that the current study design does not control for all possible confounds. We are aware that having a vocalization condition means both having the vocal production and the auditory feedback. We are also aware that acoustic features have a considerable contribution to arousal/autonomic effects.
We are aware that from our data, we cannot determine whether the observed brain effects (alpha and theta power increases) are due to auditory feedback or vocal production. This was outside of the scope of the current study. Our exploratory study rather aimed to focus on the difference between respiration contributions and the effects of respiration frequency. We take your suggestion and offer directions for future studies comparing vocal production with a controlled auditory feedback condition (see last paragraph of the discussion).

3. The authors attribute alpha/theta increases to "relaxation" or "mindfulness-like" states without including subjective measures of affect, relaxation, or mental state at those timepoints. They infer cortical relaxation via post-intervention alpha and theta power increases, yet no temporal dynamics or task-state EEG are estimated. This limits any causal or even associative interpretation of these changes. Further, the observed alpha/theta increases could reflect post-task relaxation rather than direct effects of vocalization.

The current paper does not present any results with regard to subjective states associated with slow breathing, toning, or singing. Instead, we mention that increases in alpha and theta power have been associated with subjectively experienced relaxation in previous studies. In particular, the previous report, which used the current dataset (Snow et al., 2018), provides a more in-depth

account of the phenomenological aspects of toning, breathing, and singing. It is thus outside of the scope of the current report.

Again, we are aware of the limitations of considering only the baseline before and after the intervention. However, we believe that a generic post-task relaxation effect would show the same effects across all conditions, and in Figures 3 and 4, we can appreciate that there are contrasting effects between breathing and vocal conditions. In addition, the singing and toning conditions show a distinct topographical pattern of activation. For instance, the theta power changes for toning are frontal, whereas the changes for singing and dorsal. We thus discard post-task relaxation as a possible explanation.

4. The authors treat HRV and EEG as separate outputs, yet the study's stated goal was to explore neural-autonomic coupling, which was not tested directly (e.g., via phase-amplitude coupling, Granger causality, etc.).

The goals and hypotheses of the study are presented in lines 51-66. Specifically: "The goals of this study were to describe the neural profiles associated with the slow breathing and vocal action components of toning and to test the effects of toning in parasympathetic activation". We have updated the abstract to further clarify, from "we aimed to describe the neural responses of toning and singing familiar songs with a slow rhythm of a group of participants without prior music training, in relation to their autonomic responses measured via heart rate variability (HRV)." To "we aimed to describe the neural responses to toning and singing familiar songs with a slow rhythm of a group of participants without prior music training, and their autonomic responses measured by heart rate variability (HRV)".

Since this study was part of a larger study with cardiorespiratory (Bernardi et al., 2017) and phenomenological (Snow et al., 2018) dimensions already reported, the focus of the study was the EEG. We introduced HRV-RMSSD as a marker of vagal afferent tone (see Laborde et al., 2017) to explore a possible role of this pathway in vocalization and slow breathing. Initially, there was interest in obtaining heartbeat event-related potentials (HERP) to quantify the autonomic effects in the brain (e.g., Luft & Bhattacharya, 2015 was one reference study for our methods). Unfortunately, for technical reasons, this was not possible to achieve.

5. The paper speculates about medullary afferent relay (via NTS) modulating alpha power, but without any latency analysis, ERP components, or HR-locked EEG, this remains highly speculative.

We agree that HERP would allow for a more direct estimation of heart-related activity in the brain. Your remarks support our early intuitions when exploring the datasets. Unfortunately, as

stated above, HERP was not possible to achieve due to a mismatch in the resolution of the ECG and EEG systems used.
Regarding the mechanisms mentioned, we have presented these as previous considerations made by other authors: “It is hypothesized that slow breathing influences the activity of vagus afferents by means of baroreceptors and pulmonary afferent receptors, which project into the medulla, the parasympathetic relay nucleus and subsequently to the nucleus of the solitary tract (NTS) (12)”. This refers to the paper by Laborde et al. (2017). We slightly updated the phrasing to “has been hypothesized” to dispel possible confusions.

We understand that we do not present any mechanistic evidence with regard to how slow breathing or toning may influence the brain. What we do present is a possible explanation for the absence of effect in the toning condition regarding HRV-RMSSD, proposing that two opposing forces may be at play: the arousing aspect of motor activity vs. the effect of slower breathing in toning (around 0.1Hz) compared to singing (around 0.2Hz). Future studies may help clarify how such proposed systems interact and whether they play a role at the neural level. This was beyond the scope of the present paper.

6. No behavioral or subjective data were collected to validate the hypothesized relaxation or attentional states associated with EEG changes.

We believe this has been clarified in the response to point 3. Please refer to Snow et al. (2018) for the phenomenological aspects of the interventions.

7. The matched-breathing condition uses visual feedback of prior breathing patterns, which introduces:
- Asynchronous entrainment
- Differential attentional load
- A lack of proprioceptive coupling present in real-time vocalization

We appreciate the observation. We agree that the visual feedback in the respiration-only condition introduces potential confounds. In this study, the focus was to control in the best possible form for the effects of respiration frequency. As the reviewer will understand, to account for all possible confounds is not realistic, and instead, different studies should be targeting different aspects. Taken together, all the conducted studies will serve to best understand the phenomenon. Future studies could, for instance, provide an auditory feedback condition to contrast with a vocal condition, adding motor aspects.

We would be interested in knowing if there is a better way of controlling for prior breathing patterns that does not introduce confounds. If so, we would gladly add it to the manuscript as a possible improvement for future research.

8. The conditions are not matched for motor output, auditory feedback, cognitive engagement, or emotional valence—all critical confounds.

As in the previous point raised, we acknowledge the above as limitations of our study and are open to suggestions to add for future studies focusing on toning.

9. The authors describe that theta increases during toning were anterior, and posterior theta during singing. However, these topographical patterns of alpha and theta are not studied and are not statistically compared in a meaningful way.

We reported topographical clusters that are statistically significant based on non-parametric testing. As a result, we obtained topographical patterns for alpha, beta, and theta power, using a similar approach as in Luft & Bhattacharya (2015). Could you please clarify what you mean by "the topographical patterns…not statistically compared in a meaningful way?

Regarding the localization of the clusters, following the results from the cluster permutation analysis, we found that for theta power, the clusters showing significant changes were located towards posterior locations for toning and anterior locations for singing.

10. Beta increases are noted across all conditions and linked to attention, but the interpretations are vague and speculative. The negative correlation with RMSSD is interesting but likely epiphenomenal without mechanistic support.

Increases in beta power following all interventions were observed. As discussed (l.392-404), our findings of beta power increases for all conditions align with the literature, as we would expect all experimental conditions to require attention and cognitive control, and beta power being related to these cognitive components (Lee et al., 2018 and Güntekin et al., 2013). The explanation we offer is consistent with previous literature. A more precise mechanistic approach is outside the scope of this study.

As the reviewer mentions, we found an important negative correlation between HRV-RMSSD and beta power. Beta power increases are linked to cognitive control and attention, while focused activity on a task is linked to HRV-RMSSD decreases (e.g., Melo, Nascimento & Takase, 2017). We believe our findings to be aligned with the literature. However, we agree that a clear mechanism cannot be supported by our results, as these analyses were only exploratory.

11. The use of HRV-RMSSD as a proxy for vagal tone is correct in principle, but the discrepancy between SDNN/LF and RMSSD is not deeply analyzed.

We would like to refer the reviewer to the previous article part of the larger study (Bernardi et al., 2017), which specifically targeted the cardiorespiratory patterns. A complete replication was outside the scope of the current manuscript. Rather, the goal was to validate that the previous indices (LF, SDNN) did not differ when using a smaller sample (16 participants instead of the original 20, as 4 datasets were dropped due to issues with EEG data). The current study introduced RMSSD as a well-known biomarker of afferent vagal tone, which is less influenced by respiratory frequency than LF or SDNN (Laborde et al., 2017). We explain in the article (l.204-212) that the LF index of HRV is highly dependent on the respiration frequency: a respiration frequency lower than 0.15Hz will lead to a peak in the LF range due to respiratory sinus arrhythmia. A more in-depth analysis of the differences between these measures is outside the scope of this paper.

12. The assertion that vocalization suppresses vagal tone via HR increases is plausible but not directly demonstrated. Any of the following would be relevant:
- Beat-to-beat coupling or cardio-vagal coherence
- Time-resolved measures across the intervention
- Simultaneous blood pressure/baroreflex data

We appreciate the observation made by the reviewer and agree that our findings are not conclusive with regard to the effects of vocalization on vagal tone. For practical reasons, it was not feasible to have a blood pressure monitor in this study. Future studies should consider this, as we mention in the suggestions for future work.

13. Artifact removal uses standard ICA and interpolations, but:
- No quantification of remaining noise variance
- No channel-wise SNR metrics
- No head motion capture to verify that the vocalization-induced artifact wasn't residual

We appreciate the suggestions made by the reviewer. Because the analyses were made only for resting baselines, there was no need to use any technique to control for movement during the interventions.

ICA was indeed used to remove eye blinks. Our analyses were based on standard methods and previous research that do not refer to noise variance and channel-wise SNR (Luft & Bhattacharya, 2015; Timmermann et al., 2019). Further, estimates such as SNR are also not fully reliable and must be interpreted with caution. We appreciate the observation, and at the same time, consider that these metrics are not needed.

Recommendations

- Include or recover task-state EEG data using artifact-resistant approaches to examine real-time dynamics.

We appreciate the suggestion, and these approaches can be considered for future work. We do not feel confident we can remove the artifacts, especially since they may be very different between conditions, introducing potential confounds, etc.

- Incorporate subjective measures of emotional or cognitive states to support interpretations of EEG changes.

Subjective reports from participants are already published in the 2018 paper (Snow et al., 2018). Future work may examine micro-phenomenological methodologies such as those used by Timmermann et al. (2019) and proposed by Petitmengin.

- Control or model auditory and attentional components across vocal and breathing conditions.

We appreciate this suggestion, and we will make sure to include this in the directions for future research.

- Use time-resolved analyses of EEG-HRV coupling or HR-locked ERPs to support claims of central-autonomic dissociation.

We thank this observation since it supports our intuitions regarding HERPs. This is also included in our limitations paragraph in the discussion.
- Consider alternative explanations such as physical exertion, sensory feedback, or performance relief to account for post-intervention EEG changes.

Thank you for the suggestion. We include physical exertion and sensory-motor pathways as one of the possible explanations for the differential effects observed in the EEG between vocal and non-vocal conditions. This naturally leads to directions for future research.

Reviewer 3

1. Data availability: The current data availability statement (“available upon request”) does not meet PLOS ONE’s open data policy. To proceed toward publication, the authors should deposit

the anonymized dataset in a public repository (e.g., OSF, Zenodo, Dryad) and include the DOI or permanent link in the manuscript.

We appreciate the observation regarding the data availability statement. This would be the ideal case. However, because of the ethics approval by McGill's IRB (ethics board), we are not allowed to have the data publicly on an open repository. We have added the following data availability statement:

"The data that support the findings of this study are not openly available because the participants of this study did not give written consent for their data to be shared publicly. Data are available upon reasonable request by contacting the corresponding author, the Principal Investigator, or the Faculty of Medicine and Health Sciences Research Ethics Board (McGill IRB), which approved this study."

2. Participant exclusion: Seven participants were excluded from the original sample of 23. While this is not unusual in EEG research, it would strengthen the manuscript to clarify the reasons and potential implications (e.g., on statistical power or sample representativeness).

Seven datasets were discarded. We specify the reasons in the Participants section (l.69-79).

We here reproduce the response to one of the reviewers, in the hope of bringing clarity. "It is important to note that this report, as part of a larger study (including a report on the cardio-respiratory effects and a report on the phenomenological aspects), had exploratory objectives. The adequacy of the sample size was based on prior studies tackling exploratory topics in the realm of cardiorespiratory interactions, including previous papers from our team. Some examples of studies having around 20 participants are Spicuzza et al (2000) in Lancet, Bernardi et al (2001) in BMJ, Bernardi et al (2006) in Heart, Bernardi et al (2009) in Circulation.

The sample size aligns with the recommendation for exploratory research, particularly for within-subject designs, suggesting that "increasing the sample size to 12 participants made a profound difference in the width of confidence intervals for mean response, whereas increasing the sample size beyond 12 participants did not. They referred to this result as a "rule of 12" for continuous variables. We recommend at least 12 participants for pilot studies with primary focus of estimating average values and variability for planning larger subsequent studies. This size is quite practical for most early-stage investigators to conduct within single centers while still providing valuable preliminary information" (Moore et al., 2011)".

For clarity, the following was added to the Participants' section:
"Having exploratory purposes and a within-subjects design, this study aimed to have more than twelve participants, as previously recommended (Moore et al., 2011)."

3. Justification for exploratory analyses: The inclusion of beta and low-gamma EEG bands is labeled as exploratory, but the rationale for these choices could be more thoroughly discussed or contextualized within the literature.

We appreciate the observation. We added an explanation referring to possible roles of beta and low-gamma: “Beta power is often considered to be associated with sensorimotor 177 processing, attention, emotion, and cognitive control [39, 66], which could reflect 178 attention to the task. Low gamma power may play a role in sensory processing [24] and 179 in motor processing [23]” (l.177-180).

4. Timeline clarity: A visual schematic of the experimental protocol and timing (baseline, intervention, post-intervention, etc.) would improve accessibility, especially for interdisciplinary readers.

We also believe a visual schematic to be useful in better understanding the experimental procedure. We would like to point the reviewer to Fig. 1, as perhaps it was missed (especially since the figures are presented separately from the manuscript).

5. Language and phrasing: The manuscript is well written, but a few minor edits would improve clarity (e.g., rephrasing “HRV was greater for breathing-only” to “HRV was higher during the breathing-only condition”).

We revised the whole manuscript to make sure the phrasing is clear.
