# Supplementary material for: Neural and cardiorespiratory responses in vocalization and slow breathing: Contrasting brain and autonomic responses
Source: PLoS One. 2026 Apr 29;21(4):e0345719. doi: 10.1371/journal.pone.0345719 (PMC13128120; doi:10.1371/journal.pone.0345719)
Supplement: Attachment — Submitted filename: Response_to_Reviewers_auresp_3.pdf [file pone.0345719.s004.pdf]

Response to Reviewers
12 January 2026

Dear Reviewers/Editor,

We hope this letter finds you well. On January 8, we received the following points to improve the manuscript, which we address below.

"1. Female/male ratio vs. final sample size
Please correct the apparent inconsistency between the reported female/male ratio and the final sample size (N = 16). Either provide the sex breakdown for the final analysed sample, or clearly distinguish between the sex distribution of the initial recruited sample and that of the final dataset.

2. "Afferent" vs. "efferent" vagal tone in the Abstract
In the Abstract, RMSSD is described as indexing "afferent vagal tone", whereas in the Methods it is presented more generally as a vagal HRV measure associated with parasympathetic cardiac control, typically interpreted as efferent vagal activity to the heart. I recommend revising the Abstract to refer to RMSSD as "vagal tone", "vagal activity" or "parasympathetic cardiac control", in line with the Methods and HRV guidelines.

3. Wording about discarding post-task relaxation
Your argument that condition-specific and topographical differences make a purely generic post-task relaxation explanation unlikely is reasonable. I would simply suggest softening the sentence where you "discard" this explanation, for example: "we consider a purely generic post-task relaxation explanation unlikely in light of the condition-specific and topographical patterns observed."

4. Framing of mechanistic interpretations
You already mark mechanistic interpretations (NTS, baroreflex pathways, competing influences of motor arousal and breathing frequency, etc.) as hypothetical. I encourage you to maintain this careful wording throughout the Discussion, using formulations such as "it has been hypothesized that…", "one possible explanation is…", or "our findings are consistent with the idea that…", rather than stronger causal language.

5. Clarifying the reporting scale for HRV values (optional)
If HRV values in the main tables are reported on the original (untransformed) scale, it could help readers if this is stated explicitly (e.g., "HRV values are reported on the original scale for interpretability, although analyses were conducted on transformed data").

6. Effect sizes for key results (optional)
Reporting simple effect sizes (e.g., partial η^2 for main ANOVA factors, Cohen's d for key contrasts) would further strengthen the statistical reporting and help readers gauge the magnitude of the observed effects, although I do not consider this a requirement for publication."

1. **Female/Male ratio**. We have updated the female/male ratio to 12/4, consistent with the reduced data for the current study.
2. **Efferent vs. afferent**. Because it is a feedback loop system, efferent and afferent influences may be hard to disentangle when looking at HRV, and, specifically, RMSSD. However, it is more directly coupled with the effect of the parasympathetic nervous system on the heart (efferent vagal activity). For these reasons, we decided it is best to simply make reference to vagal tone or keeping the term "efferent" when relevant.
    a. We specifically updated the following sentence for clarity: "It is possible that, by increasing HR, the vocal conditions counteract this vagal afferent feedback, resulting in no observable effect in HRV-RMSSD." to: "Unlike simple breathing, vocalizing increases physical exertion. This reduces vagal tone and typically raises the heart rate. These changes likely mask the effects of vagal afferent feedback, which on its own would cause HRV-RMSSD to rise."
    b. To avoid confusion, we removed "afferent" in several places in the Discussion.
3. **Wording about post-task relaxation**. We are keen on improving the wording to improve the readability, but are not clear what is the part of the manuscript the reviewer is refering to. *Could you please point to the exact paragraph in the text?*
4. **Framing of mechanistic interpretations**. We agree mechanistic interpretations are strictly hypothetical and this work provides little evidence in this regard. We have revised the discussion to make sure this tone is carefully maintained.
5. **Clarifying the reporting scale for HRV values**. We incorporated the reviewer's suggestion as a caption for table 1: "All values are reported on the original scale for interpretability, although analyses were conducted on transformed data"
6. **Effect sizes for key results**. We obtained the partial η^2 for main ANOVA factors and Cohen's d for direct comparisons and reported in the table. Readers may now better understand the statistical effect sizes.

We thank you again for your time and effort in making this review.

On behalf of all the authors,
Sebastián Ruiz-Blais
